# Corrigendum

**DOI:** 10.1111/jdi.13501

**Published:** 2021-03-03

The authors would like to draw the reader’s attention to the errors in the following articles.

Kaga H, Tamura Y, Takeno K, Kakehi S, Someya Y, Funayama T, Furukawa Y, Suzuki R, Sugimoto D, Kadowaki S, Nishitani‐Yokoyama M, Shimada K, Daida H, Aoki S, Giacca A, Sato H, Kawamori R, Watada H. Shape of the glucose response curve during an oral glucose tolerance test is associated with insulin clearance and muscle insulin sensitivity in healthy non‐obese men. *J Diabetes Investig* 2020; 11: 874–877.

Sato M, Tamura Y, Someya Y, Takeno K, Kaga H, Kadowaki S, Sugimoto D, Kakehi S, Funayama T, Furukawa Y, Suzuki R, Nakagata T, Nishitani‐Yokoyama M, Shimada K, Daida H, Aoki S, Sato H, Kawamori R, Watada H. Characteristics associated with elevated 1‐h plasma glucose levels during a 75‐g oral glucose tolerance test in non‐obese Japanese men. *J Diabetes Investig* 2020; 11: 1520–1523.

In both of the articles above, ‘Hiroaki Sato’ should have been spelled as ‘Hiroaki Satoh’.

The authors apologize for these errors and any confusion they may have caused.

